# Selective and ATP‐competitive kinesin KIF18A inhibitor suppresses the replication of influenza A virus

**DOI:** 10.1111/jcmm.15200

**Published:** 2020-04-06

**Authors:** Yong‐Bin Cho, Sungguan Hong, Kyung‐Won Kang, Ji‐Hun Kang, Sang‐Myeong Lee, Young‐Jin Seo

**Affiliations:** ^1^ Department of Life Science Chung‐Ang University Seoul South Korea; ^2^ Department of Chemistry Chung‐Ang University Seoul South Korea; ^3^ Division of Biotechnology College of Environmental and Bioresources Jeonbuk National University Iksan South Korea

**Keywords:** influenza virus, KIF18A, RanBP3

## Abstract

The influenza virus is one of the major public health threats. However, the development of efficient vaccines and therapeutic drugs to combat this virus is greatly limited by its frequent genetic mutations. Because of this, targeting the host factors required for influenza virus replication may be a more effective strategy for inhibiting a broader spectrum of variants. Here, we demonstrated that inhibition of a motor protein kinesin family member 18A (KIF18A) suppresses the replication of the influenza A virus (IAV). The expression of KIF18A in host cells was increased following IAV infection. Intriguingly, treatment with the selective and ATP‐competitive mitotic kinesin KIF18A inhibitor BTB‐1 substantially decreased the expression of viral RNAs and proteins, and the production of infectious viral particles, while overexpression of KIF18A enhanced the replication of IAV. Importantly, BTB‐1 treatment attenuated the activation of AKT, p38 MAPK, SAPK and Ran‐binding protein 3 (RanBP3), which led to the prevention of the nuclear export of viral ribonucleoprotein complexes. Notably, administration of BTB‐1 greatly improved the viability of IAV‐infected mice. Collectively, our results unveiled a beneficial role of KIF18A in IAV replication, and thus, KIF18A could be a potential therapeutic target for the control of IAV infection.

## 1 INTRODUCTION

The influenza virus is a major cause of serious respiratory infections, with systemic manifestations that include fever, coughs, diarrhoea and headaches, and it is associated with significant morbidity and mortality.^1^, ^2^, ^3^ Despite vaccination, 5%‐15% of the population suffer from influenza virus infections annually due to frequent mutations in the viral RNA genome.^4^ Moreover, influenza pandemics occur once every several decades. For example, the swine influenza (H1N1) pandemic in 2009 led to 151 000 to 575 000 deaths globally,^5^ which has continuously raised concerns about the emergence of next potential influenza pandemic.^6^, ^7^ Although antiviral drugs such as oseltamivir and amantadine are currently available, multiple strains that exhibit resistance to these drugs have been reported.^8^, ^9^, ^10^, ^11^, ^12^ Thus, the development of novel therapeutics that can protect the host from a broad range of influenza variants is needed.

The influenza virus is a member of the *Orthomyxoviridae* family, and its genome consists of eight single‐strand RNA segments, encoding 11‐12 proteins.^13^ The influenza virus life cycle is divided into multiple steps including entry, replication of the viral genomic RNAs, export of viral ribonucleoprotein (vRNP) complexes from the nucleus, assembly and release.^14^ Each step in this process strongly depends on specific interactions with host cellular elements. For example, PI3K/AKT/mTOR signalling pathways are activated during viral entry,^15^ and the transcription and replication of viral RNAs are also known to require the activation of NF‐κB.^16^ In addition, Raf/MEK/ERK/NF‐κB signalling pathways are involved in the nuclear export of vRNP complexes and release processes.^17^, ^18^ Although inhibiting these signalling pathways could block influenza virus replication, they are essential to host cell survival as well. Therefore, it is important to discover host cell factors that regulate influenza viral replication, while their inhibition minimally affects the host cells.

Viruses use the cytoskeleton transport system during their replication cycle, with interactions between subviral molecules and cytoskeleton proteins critical for virus assembly and release.^19^, ^20^, ^21^, ^22^, ^23^, ^24^ A kinesin is a motor protein that moves various substances such as diverse membranous organelles, mRNAs, intermediate filaments and signalling molecules within cells along microtubules.^25^ Kinesins are known to play a role in regulating cell division, cell movement, spindle assembly and chromosome alignment/segregation.^26^, ^27^, ^28^ Interestingly, it has been recently reported that kinesin family member proteins regulate the replication of some viruses, including HIV,^29^ HSV^30^ and the Lassa virus.^31^ Thus, kinesin family proteins could be potential antiviral targets. KIF18A is a member of the kinesin motor protein family and known to play a critical role in diverse cellular processes including microtubule dynamics and subcellular organelle transportation.^32^ While KIF18A is known to be associated with several cancers,^33^, ^34^, ^35^ no role has been previously described for this protein in viral infection.

In this study, we investigated the role of KIF18A in the replication of the influenza A virus (IAV). We discovered that KIF18A is a beneficial host protein for IAV replication. Importantly, the inhibition of KIF18A by a highly specific small‐molecule inhibitor, BTB‐1,^36^, ^37^, ^38^ significantly suppressed IAV replication and enhanced the survival rate of IAV‐infected mice. Thus, KIF18A could be targeted as a potential therapy to control IAV infection.

## 2 METHODS

### 2.1 Virus, cells and infection

Influenza *A/California/04/2009* (H1N1) virus was kindly provided by Dr Baik Lin Seong (Yonsei University). Amplification and titration of the virus were performed on Madin‐Darby Canine Kidney (MDCK) cells as described previously.^39^ MDCK cells were maintained in Eagle's minimum essential medium (MEM, Welgene) supplemented with penicillin/streptomycin (Welgene) and 10% foetal bovine serum (FBS, HyClone). Human embryonic kidney (HEK) 293 cells and human lung epithelial (A549) cells were maintained in Dulbecco's modified Eagle's medium (DMEM, Welgene) supplemented with penicillin/streptomycin and 10% FBS. In order to infect the cells, MDCK, HEK293 or A549 cells were incubated for 1 hour with the influenza virus in the presence of MEM or DMEM (Welgene) containing 0.3% bovine serum albumin (BSA) and TPCK‐trypsin (2 μg/mL, Sigma‐Aldrich).

### 2.2 Reagents, plasmids and antibodies

A KIF18A inhibitor (BTB‐1, ≥99%) was purchased from Tocris Bioscience. To treat the infected cells with BTB‐1, cells were washed with PBS twice after 1‐hr incubation with IAV to wash out unbound virus. For KIF18A overexpression, plasmid DNA (pMX229 was a gift from Linda Wordeman, Addgene plasmid #23002) was used.^40^ The transfection of cells with the plasmids was performed using Lipofectamine™ 3000 Reagent (Invitrogen) according to the manufacturer's instructions. Antibodies used to detect influenza viral M1, NP and KIF18A were purchased from Abcam. Anti‐influenza viral NS1 was purchased from Santa Cruz Biotechnology. Antibodies against actin, AKT, p‐AKT (Ser473), p38, p‐p38, SAPK, p‐SAPK, RanBP3 and p‐RanBP3 were purchased from Cell Signaling Technology. Anti‐rabbit IgG, HRP‐linked antibody was purchased from Cell Signaling Technology. Goat antimouse IgG F(ab')2, polyclonal antibody (HRP conjugate) was purchased from Enzo Life Sciences.

### 2.3 Trypan blue exclusion assay

Cell viability was analysed by a trypan blue exclusion assay as described previously.^41^ Briefly, 50 μL of cell suspension was mixed with equal parts of 0.4% trypan blue dye (Thermo Scientific). After incubation for 1 minute, cells were counted under a light microscope. Cell viability was calculated by dividing the number of viable cells (unstained) by the number of total cells and multiplying by 100.

### 2.4 Western blot analysis

Western blotting was performed as described previously.^39^, ^42^ All proteins were extracted using an NP 40 protein extraction solution (Elpisbio) supplemented with a protease/phosphatase inhibitor (Thermo Scientific). Amount of protein was quantified using Pierce BCA protein assay kit according to the manufacturer's instruction (iNtRON Biotechnology). Equal amounts (10‐20 μg) of protein were loaded onto SDS‐PAGE gels (10% or 12% or 15%) for separation before being transferred onto nitrocellulose membranes. After incubating the membranes with primary antibodies (1:1,000) overnight at 4°C, membranes were incubated with the secondary antibody (1:5000) at room temperature for 1 hr Membrane‐bound antibodies were detected using SuperSignal West Pico PLUS‐enhanced chemiluminescent substrate (Thermo Scientific).

### 2.5 Flow cytometric analysis

To detect intracellular influenza viral proteins, cells were permeabilized using a Foxp3/transcription factor staining kit (Tonbo Biosciences) according to the manufacturer's instructions. Briefly, after fixation and permeabilization of the cells, anti‐influenza viral M1 (5 µg/mL, Abcam) or NP antibodies (2.5 µg/mL, Abcam) were added, followed by staining with Alexa fluor 488‐conjugated secondary antibodies (10 µg/mL, Thermo Scientific) or Alexa fluor 594‐conjugated secondary antibodies (10 µg/mL, Abcam). Data were collected using an Attune™ NxT Acoustic Focusing Cytometer (Thermo Scientific) and analysed using a FlowJo software (BD Biosciences).^43^

### 2.6 Plaque assay

For IAV titration, MDCK cells were plated onto 6‐well plates. On the following day, the cells were incubated with supernatant containing IAV. After incubation for 1 hour, the infected cells were overlaid with 1% SeaPlaque agarose diluted in MEM containing 0.3% BSA. Three days later, the cells were fixed with 25% formaldehyde solution. The cells were stained with 1% crystal violet (Sigma) dye solution for 10 minutes and rinsed with PBS three times to remove excess dye. The plaques are readily visible where the cells have destroyed by the infection.

### 2.7 RNA interference

Small interfering RNA (siRNA) targeting KIF18A and scramble siRNA were purchased from Thermo Scientific. The knock‐down of KIF18A with a specific siRNA was conducted using Lipofectamine® RNAiMAX Reagent (Invitrogen) according to the manufacturer's instructions. Briefly, HEK293 cells that were seeded in 24‐well plate one day earlier were treated with Lipofectamine RNAiMAX reagent/siRNA (si‐KIF18A or scramble siRNA) mixtures for 24 hours. RNA interference efficiency was analysed by Western blotting.

### 2.8 Immunofluorescence analysis

Cells were plated on a 4‐well cell culture chamber slide (Millipore) and infected with IAV at multiplicity of the infection (MOI) of 5 for the indicated time. After permeabilization and fixation, the cells were incubated with anti‐influenza viral NP antibodies (1 μg/mL; Abcam), followed by incubation with Alexa fluor 488‐conjugated secondary antibodies (Thermo Scientific). The cells were mounted with Fluoroshield‐containing DAPI (Thermo Fisher). Images were obtained using an LSM710 (Carl Zeiss) confocal microscope at 400× magnification and analysed using LSM Image Browser (Carl Zeiss). For each slide, at least three fields were visualized.

### 2.9 Real‐time quantitative PCR

HEK293 cells were infected with IAV at MOI of 3 for 3 hours or 6 hours. Total RNA was extracted using RNAiso Plus reagent (Takara) to synthesize the cDNA using ReverTraAce qPCR RT kit (Toyobo). The quantitative real‐time PCR for viral RNA (M1, NP, HA) was performed using a CFX Connect real‐time system (Bio‐Rad). The following primers were used: GAPDH forward, 5’‐TGGACCTGACCTGCCGTCTA‐3’, reverse 3’‐CCCTGTTGCTGTAGCCAAATTC‐3’ and M1 forward 5’‐AAGACCAATCCTGTCACCTCTG‐3’, reverse 5’‐CAAAACGTCTACGCTGCAGTCC‐3’ and NP forward 5’‐CCAGATCAGTGTGCAGCCTA‐3’, reverse, 5’‐CTTCTGGCTTTGCACTTTCC‐3’ and HA forward 5’‐GGCCACAGGATTGAGGAATA‐3’, reverse 5’‐TGGCATTCTGTGTGCTCTTC‐3’. The threshold cycle number (CQ) for each sample was determined in triplicate and normalized against GAPDH.

### 2.10 Animals

Six‐ to eight‐week‐old male C57BL/6 mice were used in experiments. All mice were bred and maintained in a closed breeding facility and transferred to an Animal Biosafety Level 2 (ABSL2) facility of the Korea Zoonosis Research Institute (KoZRI) for IAV infection. All experiments were performed according to the protocol approved by the Institutional Animal Care and Use Committee at Jeonbuk National University (CBNU 2019‐013). For infection, 6‐ to 8‐week‐old C57BL/6 mice were infected intranasally with 1.5 × 10^3^ PFU/mouse. At 10 mpi (minutes post‐infection), 3 hpi (hours post‐infection) and 6 hpi, mice were intranasally treated with DMSO or BTB‐1 solution (3 mg/kg). Bodyweight was measured daily for 17 days.

### 2.11 Statistical analysis

All statistical analyses were performed using GraphPad Prism 5 software (GraphPad Software). Error bars indicate the standard error of the mean (SEM), and mean values were compared using Student's *t* test or ANOVA followed by Tukey's post hoc test. *P* value for mouse mortality was calculated using a log‐rank test. All experiments were repeated independently at least three times.

## 3 RESULTS

### 3.1 IAV infection increases the expression of KIF18A

**Figure 1:**
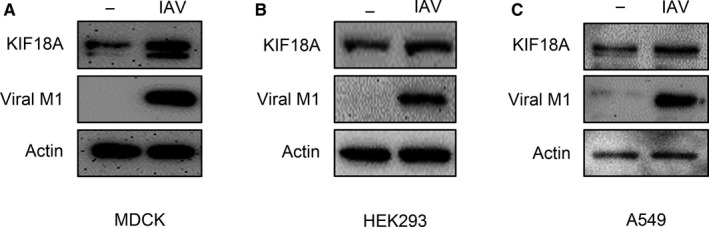
IAV infection increases KIF18A expression. (A) MDCK cells were either uninfected or infected with IAV at an MOI of 5 for 10 h. (B) HEK293 cells were either uninfected or infected with IAV at an MOI of 0.1 for 12 h. (C) A549 cells were either uninfected or infected with IAV at MOI of 3 for 12 h. Cell lysates were prepared for Western blot analysis to detect KIF18A, viral M1 and actin. Representative data of at least three independent experiments are shown

During IAV replication, diverse viral components such as viral proteins and RNAs are transported to specific sites within a host cell. Thus, it is possible that host cellular motor proteins such as kinesins are required for IAV propagation. This prompted us to investigate the role of KIF18A, a member of the kinesin motor protein family, in IAV propagation. We first measured the expression level of KIF18A in the infected MDCK cells (Figure 1A), HEK 293 cells (Figure 1B) and A549 cells (Figure 1C). After infection (as monitored by M1 expression), KIF18A expression increased in all three cell lines. These results suggest that KIF18A might play a role in IAV replication.

### 3.2 Treatment with a KIF18A inhibitor reduces the IAV‐mediated cytopathic effect

**Figure 2:**
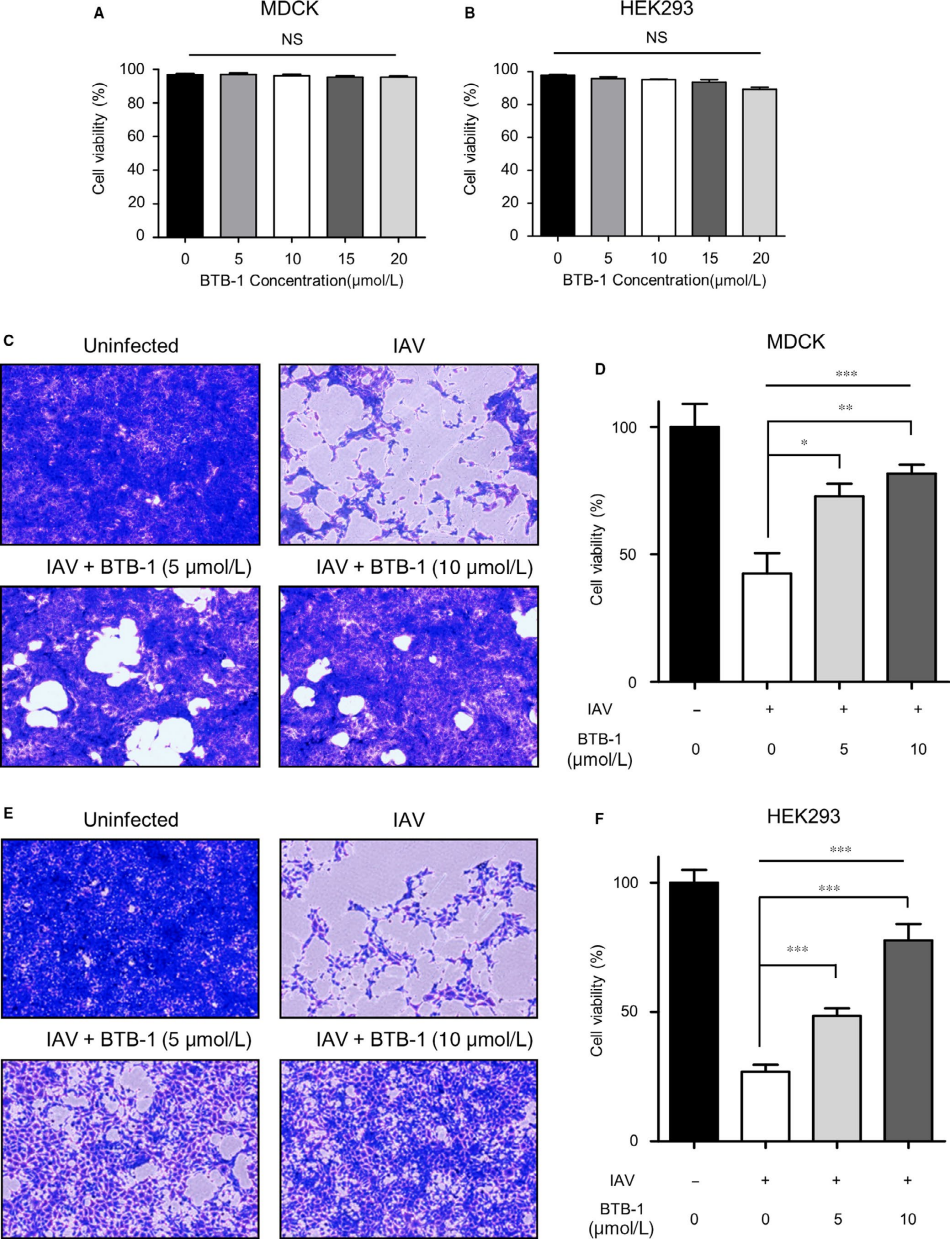
KIF18A inhibitor treatment reduces the IAV‐induced cytopathic effect. (A and B) MDCK (A) or HEK293 (B) cells were treated with vehicle (DMSO) or BTB‐1 (0, 5, 10, 15 or 20 μmol/L). Cells were harvested at 24 (MDCK) or 48 h (HEK293) after treatment to measure their viability using a trypan blue dye exclusion test (n = 5 for each condition). (C and D) MDCK cells were either uninfected or infected with IAV at MOI of 0.1 for 12 h (C) or MOI of 3 for 15 h (D) in the presence or absence of BTB‐1 (5 or 10 μmol/L). (E and F) HEK293 cells were uninfected or infected with IAV at MOI of 1 for 12 h (E) or MOI of 3 for 24 h (F) in the presence or absence of BTB‐1 (5 or 10 μmol/L). Cells were stained with 1% crystal violet after fixation (C and E). The number of viable cells was measured using a trypan blue dye exclusion test (n = 5 for each condition) and normalized to vehicle‐treated, vehicle‐uninfected condition. The bar graphs represent the average of 5 replicates ± SEM. NS: not significant; *P ≤ .05; **P ≤ .01; ***P ≤ .001. Representative data of at least three independent experiments are shown

Because IAV infection increased KIF18A expression (Figure 1), we subsequently tested whether the inhibition of KIF18A has any impact on virus propagation. In order to block KIF18A activity, the highly specific small‐molecule inhibitor BTB‐1 was used.^36^, ^37^ We first determined whether BTB‐1 is cytotoxic for MDCK, HEK293 and A549 cells by treating the cells with various concentrations of BTB‐1 (5, 10, 15 or 20 μmol/L) and assessing their viability. While we did not observe a significant reduction in the viability of MDCK cells with the tested concentrations of BTB‐1 (Figure 2A), a slight decrease in viability was observed when HEK293 cells were treated with a concentration of 20 μmol/L (Figure 2B). Similarly, BTB‐1 did not significantly decrease viability of A549 cells at the tested concentrations (Figure S1). Thus, we decided to use a concentration of BTB‐1 lower than 20 μmol/L in subsequent experiments. Because IAV is a cytolytic virus, the cytopathic effect (CPE) is strong evidence of infection. As expected, when MDCK (Figure 2C and D) or HEK 293 (Figure 2E and F) cells were infected with IAV, a significant CPE was observed. Interestingly, however, BTB‐1 treatment (5 or 10 μmol/L) dramatically reduced the infection‐mediated CPE in both MDCK and HEK293 cells in a dose‐dependent manner. Similarly, BTB‐1 treatment prevented IAV‐induced death of A549 cells (data not shown). These results suggest that the BTB‐1 treatment protects cells from IAV infection‐mediated CPE in vitro.

### 3.3 KIF18A inhibitor treatment decreases IAV propagation

**Figure 3:**
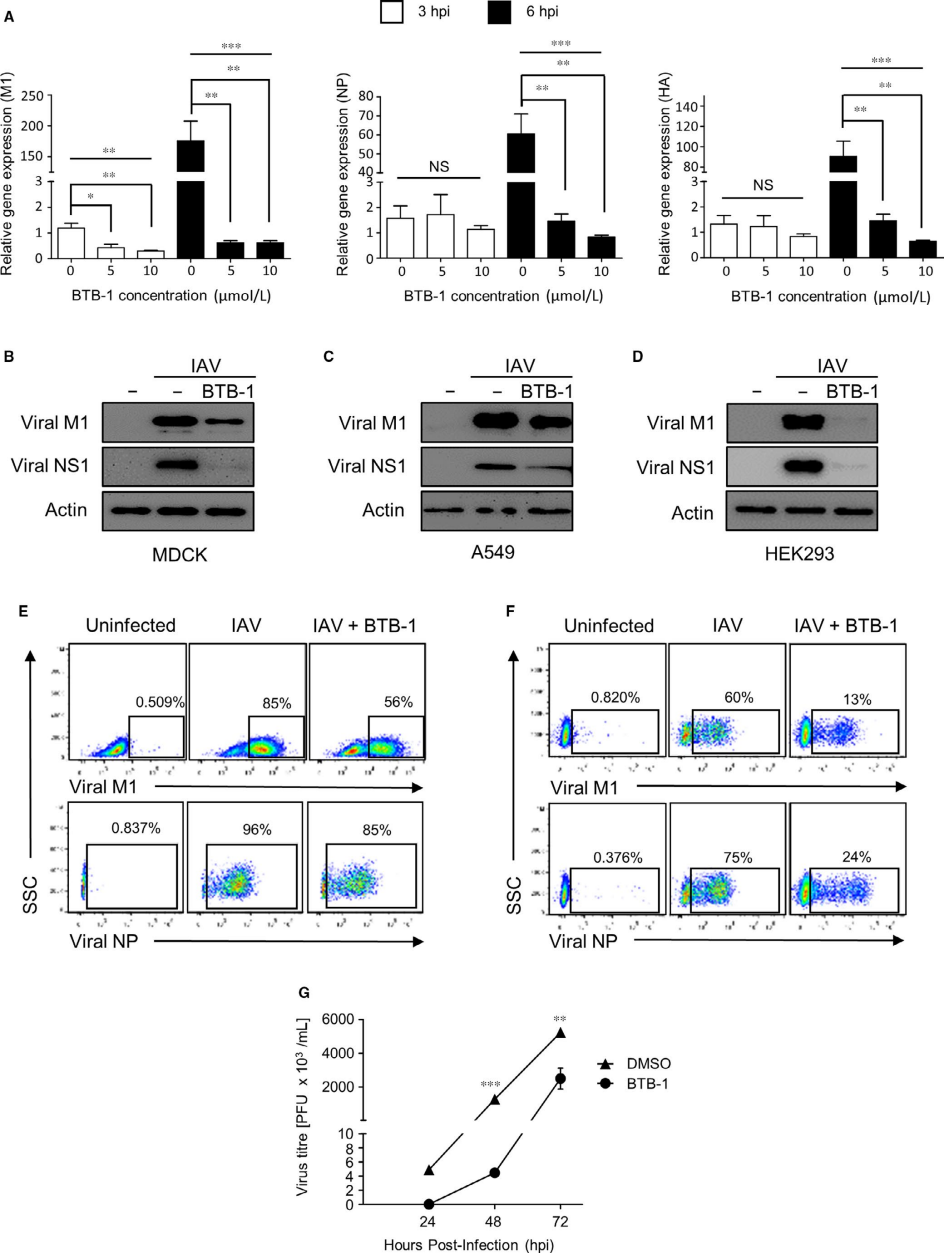
KIF18A inhibitor treatment suppresses IAV propagation. (A) HEK293 cells were infected with IAV at MOI of 3 in the absence (DMSO) or presence of BTB‐1 (5 or 10 μmol/L). At 3 or 6 hpi, cells were harvested for real‐time quantitative PCR to detect viral M1, NP and HA RNAs (n = 3 for each condition). RNA levels at 3 h post‐infection without BTB‐1 were set as 1.0. (B ‐ D) MDCK (B), A549 (C) or HEK293 (D) cells were infected with IAV at MOI of 3 (B), 5 (C) or 3 (D) for 8 (B), 12 (C) or 24 (D) hrs, respectively, in the presence or absence of BTB‐1 (10 μmol/L). Cell lysates were prepared for Western blotting to detect viral M1, NS1 and actin proteins. (E) MDCK cells were either uninfected or infected with IAV at MOI of 3 in the presence or absence of BTB‐1 (10 μmol/L). Cells were then harvested at 6 hpi or 12 hpi to detect viral NP or M1‐expressing cells using a flow cytometer. (F) HEK293 cells were either uninfected or infected with IAV at MOI of 3 in the presence or absence of BTB‐1 (10 μmol/L). Cells were harvested at 24 hpi to analyse the percentage of viral M1 or NP‐expressing cells using a flow cytometer. (G) MDCK cells were infected with IAV at an MOI of 0.001 for 24, 48 or 72 h in the presence or absence of BTB‐1 (10 μmol/L). The virus titre was measured with plaque assays (n = 3 for each condition) The graphs (A and G) represent the average of 3 replicates ± SEM. NS: not significant; *P ≤ .05; **P ≤ .01; ***P ≤ .001. Representative data of at least three independent experiments are shown

The reduced CPE following treatment with BTB‐1 (Figure 2) might represent a lower influenza virus replication rate. To test this hypothesis, we evaluated the expression of viral RNAs using RT‐qPCR. HEK293 cells were infected with IAV in the presence or absence of BTB‐1 for 3 or 6 hours. There was a strong increase in the expression of viral M1 (6 hours, 175‐fold), NP (6 hours, 60.5‐fold) and HA (6 hours, 90‐fold) RNAs following IAV infection. However, BTB‐1 treatment (5 or 10 μmol/L) was potent in preventing this increase in viral RNAs (Figure 3A). We also observed that BTB‐1 treatment decreased expression of viral RNAs in IAV‐infected MDCK and A549 cells (data not shown). Consistent with these results, a notable decrease in viral protein expression (M1 and NS1) was observed when MDCK (Figure 3B), A549 (Figure 3C) or HEK293 (Figure 3D) cells were treated with BTB‐1.

We confirmed these results using flow cytometric analysis. MDCK (Figure 3E) or HEK293 (Figure 3F) cells were treated with BTB‐1 upon IAV infection and then intracellularly stained for viral M1 or NP. Similar to our observations (Figure 3B‐D), frequencies of viral protein‐expressing cells decreased with BTB‐1 treatment. We also examined whether BTB‐1 treatment affected the production of infectious viral particles. MDCK cells were infected and then either left untreated or treated with BTB‐1 for 24, 48 or 72 hours. The supernatant was harvested to determine the viral titre using a plaque assay. The significant inhibition of infectious virus production was observed when the cells were treated with BTB‐1 (Figure 3G). Collectively, these results indicate that the inhibition of KIF18A activity interferes with IAV propagation.

### 3.4 Knock‐down of KIF18A decreases IAV replication, while its overexpression increases viral protein expression

**Figure 4:**
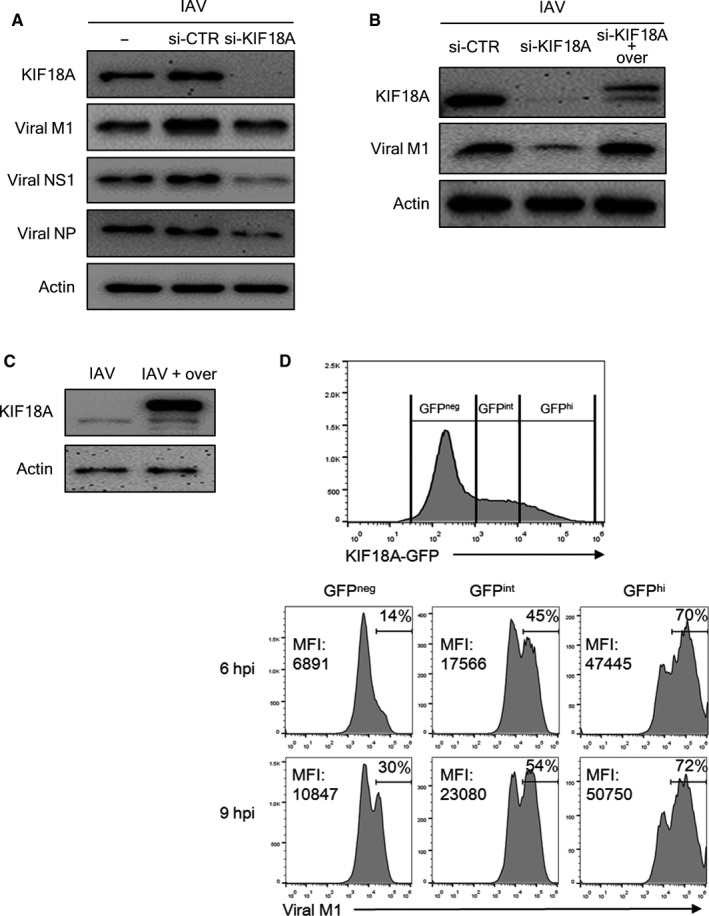
Knock‐down of KIF18A decreases IAV replication, while its overexpression increases IAV replication. (A) HEK293 cells were untransfected or transfected with scramble siRNA (si‐CTR, 5 nmol/L) or siRNA (5 nmol/L) that targets KIF18A (si‐KIF18A). After 24 h, the cells were infected with IAV at MOI of 1 for 24 h. The expression of KIF18A, viral M1, NS1, NP and actin was measured using Western blotting analysis. (B) HEK293 cells were transfected with scramble siRNA (si‐CTR, 5 nmol/L) or siRNA (5 nmol/L) that targets KIF18A (si‐KIF18A). At 24 h post‐transfection, the cells were transfected with pMX229 plasmid to express KIF18A‐GFP (over). After 24 h, the cells were infected with IAV (MOI of 1) for 18 h. The expression of KIF18A, viral M1 and actin was analysed by Western blotting. (C and D) HEK293 cells were transfected with the plasmid pMX229 to overexpress KIF18A‐GFP (over). After 24 h, the cells were infected with IAV at MOI of 3 for 24 h. (C) The expression of KIF18A and actin was analysed by Western blotting analysis. (D) The expression of GFP and viral M1 was analysed using flow cytometry. (Upper) Gates were used to define GFP‐negative (GFP^neg^), GFP‐intermediate (GFP^int^) and GFP‐high (GFP^hi^) population. (Lower) The frequency and mean fluorescence intensity (MFI) for viral M1 expression were shown. Representative data of at least three independent experiments are shown

To further confirm our observations that the inhibition of KIF18A reduces IAV replication, we utilized siRNA to target KIF18A (si‐KIF18A). The transfection of HEK293 cells with si‐KIF18A dramatically decreased the expression of KIF18A protein. Consequently, the expression of the viral M1, NS1 and NP proteins was greatly reduced (Figure 4A). We further examined whether this reduced expression of viral proteins is recovered when KIF18A is re‐expressed in KIF18A‐knock‐down cells. HEK293 cells were transfected with si‐Control or si‐KIF18A for 24 hours, and then, cells were transfected with the plasmid DNA‐encoding KIF18A. Twenty‐four hours later, HEK293 cells were infected with IAV for 18 hours. As the expression of KIF18A‐GFP fusion protein increased (Figure 4B; upper bands: exogenous KIF18A‐GFP; lower bands: endogenous KIF18A), the expression of viral M1 also increased. These results indicated that the drop in viral replication following BTB‐1 treatment was not due to non‐specific effects of BTB‐1.

As the inhibition of KIF18A decreased IAV propagation, it was considered likely that KIF18A is beneficial for IAV replication. To test this idea, cells overexpressing KIF18A were infected with IAV and viral protein levels were subsequently assessed. Transfecting HEK293 cells with plasmid DNA that encoded KIF18A^40^ resulted in a sharp increase in KIF18A‐GFP fusion protein levels (Figure 4C; upper bands: exogenous KIF18A‐GFP; lower bands: endogenous KIF18A). These cells were then infected with IAV for 6 or 9 hours. Viral M1 expressions in these cells were analysed according to KIF18A‐GFP expression level using flow cytometry (Figure 4D). Compared to GFP‐negative population (GFP^neg^), GFP‐expressing population expressed higher level of viral M1 at 6 (MFI, GFP^neg^: 6891; GFP^int^: 17 566; GFP^hi^: 47 455) or 9 hours post‐infection (hpi) (MFI, GFP^neg^: 10 847; GFP^int^: 23 080; GFP^hi^: 50 750). Similarly, frequencies of M1‐expressing population were positively correlated with expression level of KIF18A‐GFP at 6 (GFP^neg^: 14%; GFP^int^: 45%; GFP^hi^: 70%) or 9 hpi (GFP^neg^: 30%; GFP^int^: 54%; GFP^hi^: 72%). These results collectively suggest that KIF18A is a critical factor regulating viral protein expression.

### 3.5 KIF18A inhibition blocks the export of viral RNP complexes from the nucleus

**Figure 5:**
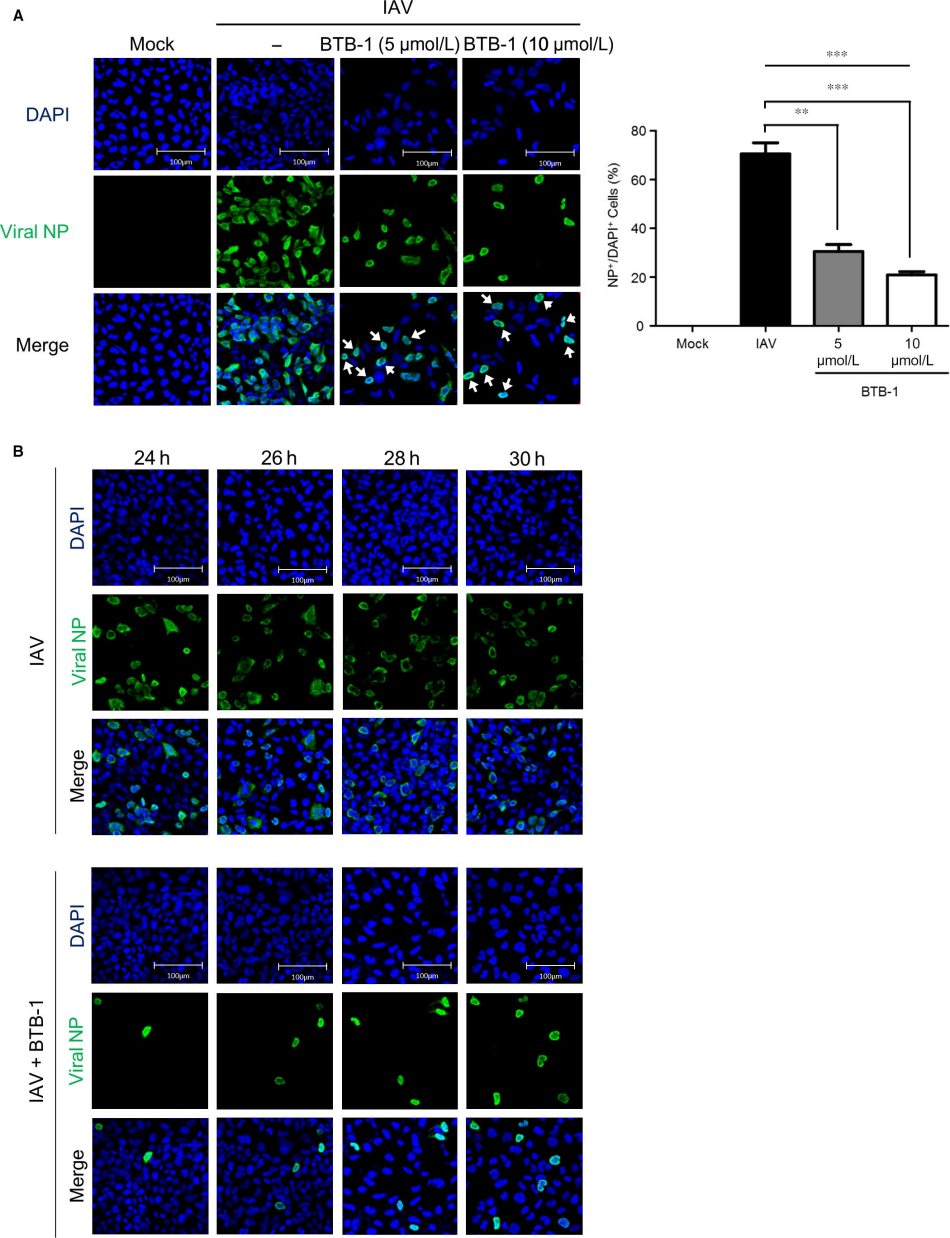
Inhibition of KIF18A activity attenuates the nuclear export of influenza vRNP complexes. (A) HEK293 cells were uninfected or infected with IAV at MOI of 5 in the presence or absence of BTB‐1 (5 or 10 μmol/L) for 30 h. Viral NP (green) and nuclei (DAPI, blue) were visualized using a confocal laser scanning microscope (left). A graph for NP+/DAPI + cells is shown (right, n = 5 for each condition) (B) HEK293 cells were uninfected or infected with IAV at MOI of 5 and treated with BTB‐1 at 5 or 10 μmol/L for 24, 26, 28 or 30 h. The data (A, right panel) represent the average of 5 replicates ± SEM. **, *P* ≤ .01; ***, *P* ≤ .001. Representative data of at least three independent experiments are shown

We conducted immunofluorescence analysis to visualize the decrease in IAV propagation caused by KIF18A inhibition. A strong increase in the number of viral NP^+^ cells (green) was observed when the cells were infected with IAV for 30 hours, while no NP^+^ cells were present in the uninfected control samples (Figure 5A). However, treatment with 5 or 10 μmol/L of BTB‐1 dramatically decreased the number of viral NP^+^ cells (Figure 5A, right), providing supporting evidence for the strong inhibitory activity of BTB‐1 on IAV propagation. Viral NP is a core component of the vRNP complex, whose export from the nucleus is a critical step in late‐phase IAV replication. Interestingly, viral NP was found to be localized in the nucleus (white arrow) after BTB‐1 treatment, while the nuclear localization of viral NP was uncommon in the untreated condition. To further confirm these results, the intracellular location of viral NP was analysed over a broader infection time frame. Similar to the results presented in Figure 5A, BTB‐1 treatment greatly reduced the number of NP^+^ cells at all time‐points. More importantly, nuclear retention of viral NP following BTB‐1 treatment was observed at all time‐points post‐infection (24‐30 hpi; Figure 5B). This indicates that the inhibition of KIF18A prevents the nuclear export of vRNP complexes.

### 3.6 Inhibition of KIF18A prevents the activation of MAPK and AKT pathways to block phosphorylation of RanBP3

**Figure 6:**
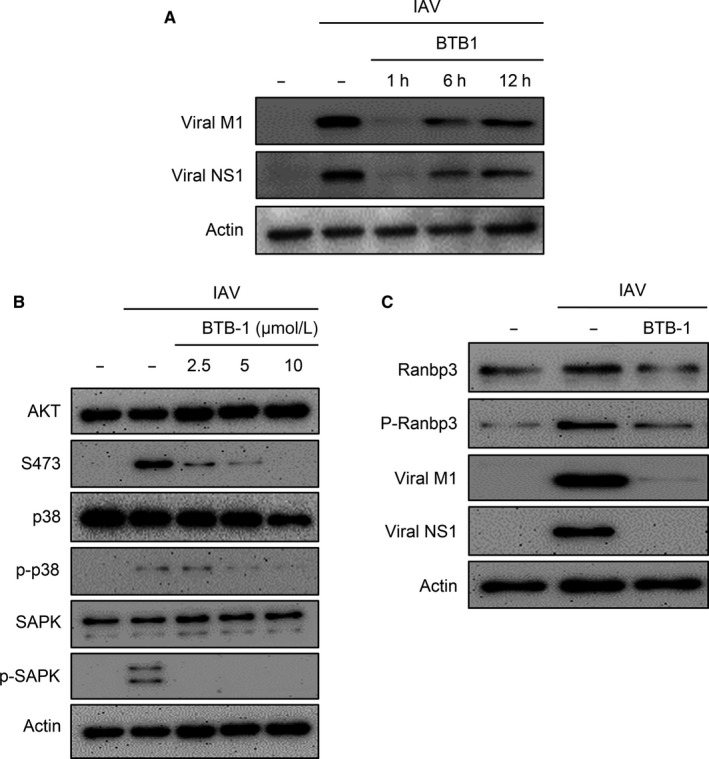
Inhibition of KIF18A prevents the activation of MAPK and AKT pathways to block the phosphorylation of RanBP3. (A) HEK293 cells were uninfected or infected with IAV at MOI of 10 and treated with BTB‐1 (0 or 10 μmol/L) at 1, 6 or 12 hpi. The cells were harvested at 24 hpi for Western blot analysis to detect viral M1, NS1 and actin. (B) HEK293 cells were uninfected or infected with IAV at MOI of 10 and treated with BTB‐1 (0, 2.5, 5 or 10 μmol/L). The cells were harvested at 24 hpi to detect AKT, p‐AKT (S473), p38, p‐p38, SAPK, p‐SAPK and actin using Western blotting analysis. (C) HEK293 cells were either uninfected or infected with IAV at MOI of 5 for 32 h in the presence or absence of BTB‐1 (10 μmol/L). Cell lysates were prepared for Western blot analysis to detect RanBP3, p‐RanBP3, viral M1, viral NS1 and actin. Representative data of at least three independent experiments are shown

We next sought to determine whether BTB‐1 also displays antiviral activity after viral entry. Following incubation with IAV for 1 hour, cells were washed to remove non‐adherent virus particles. At 1, 6 or 12 hours after the infection, the cells were treated with BTB‐1. At 24 hpi, these cells were harvested to detect viral proteins using Western blot analysis. BTB‐1 treatment at 1 hpi strongly blocked the expression of viral M1 and NS1. Interestingly, BTB‐1‐treated cells exhibited lower expression levels of viral proteins than untreated cells even at 6 or 12 hpi (Figure 6A). These results demonstrated that the inhibition of KIF18A impaired IAV propagation even after viral absorption.

The nuclear export of influenza vRNP complexes has been shown to be impaired by the inhibition of chromosome region maintenance (CRM1) activity.^39^, ^44^ The activation of AKT,^45^ SAPK/JNK ^46^ and P38 ^47^ is also known to be necessary for the CRM1‐mediated export of proteins. Because BTB‐1 blocked the nuclear export of vRNP complexes (Figure 5), we examined whether the activation of these signalling pathways was affected by BTB‐1 treatment following IAV infection. Although the phosphorylation of AKT, p38 and SAPK increased due to IAV infection, BTB‐1 strongly prevented the phosphorylation of these signalling proteins in a dose‐dependent manner (Figure 6B). In addition, BTB‐1 blocked the IAV‐induced phosphorylation of RanBP3, which is a cofactor required for the CRM1‐mediated nuclear export of vRNP complexes^39^ (Figure 6C). Collectively, these results indicate that the inhibition of KIF18A blocked the activation of AKT, p38, SAPK and RanBP3, leading to the prevention of the nuclear export of vRNP complexes.

### 3.7 KIF18A inhibitor treatment reduces the morbidity and mortality of IAV‐infected mice

**Figure 7:**
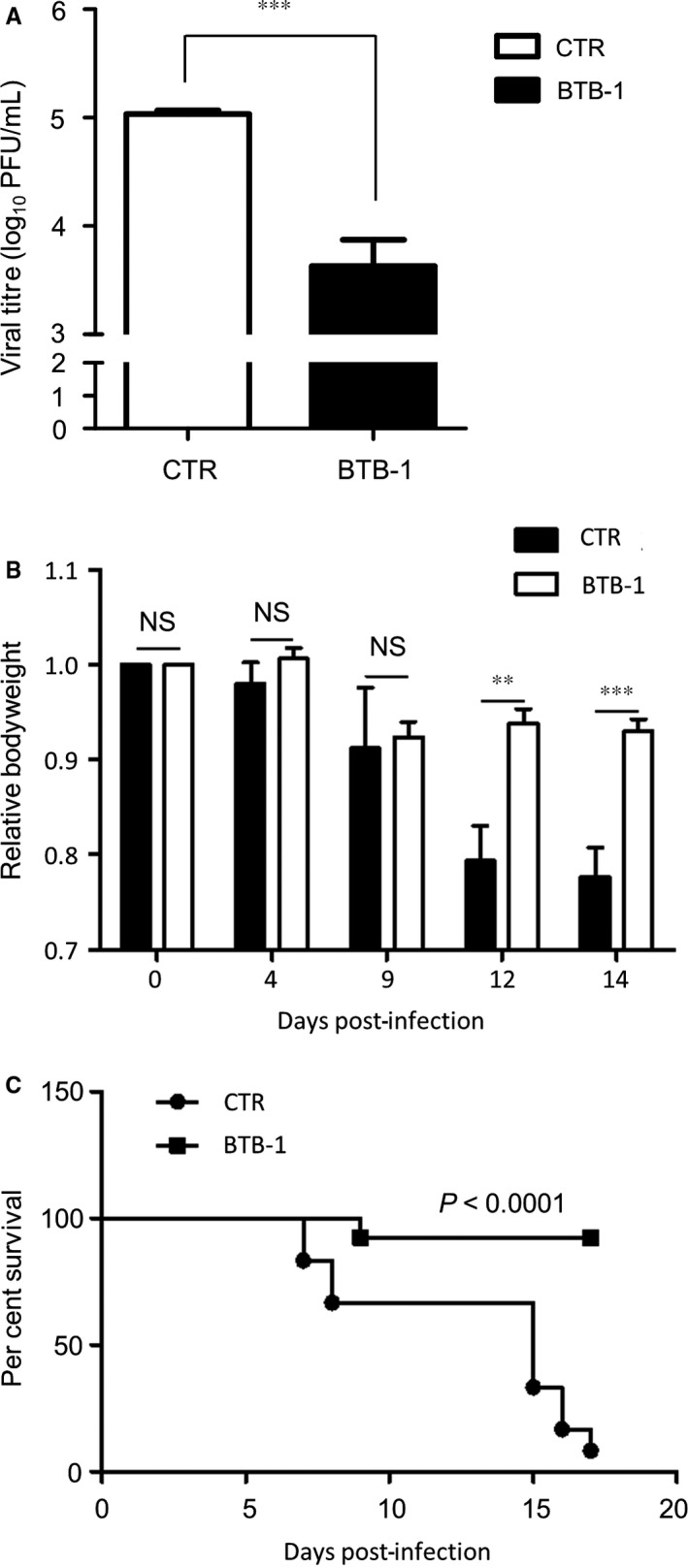
KIF18A inhibitor treatment enhances the viability of IAV‐infected mice Wild‐type C57BL/6 mice were infected intranasally with IAV at 1.5 × 10^3^ PFU. At 10 min, 3 h and 6 h after infection, mice were treated intranasally with PBS (CTR) or BTB‐1 at 3 mpk (mg per kg). (A) At 3 dpi, lungs were harvested and viral titres were determined by plaque assay. White bar indicates PBS‐treated group (CTR) (n = 5), while black bar indicates BTB‐1‐treated group (n = 5). (B) Relative bodyweights that were measured at 0, 4, 9, 12 and 14 dpi are shown. (C) Per cent survival of mice is depicted for each group. Black square indicates BTB‐1‐treated group (n = 12), while black circle indicates PBS‐treated group (CTR) (n = 12). *P* value was obtained using a log‐rank test. The bar graphs (A and B) represent the average of 5 replicates ± SEM. **P ≤ .01. ***P ≤ .001. Representative data of at least three independent experiments are shown

Based on the in vitro experiment results, we hypothesized that KIF18A inhibition could protect mice from morbidity and mortality associated with IAV infection. To test this hypothesis, we conducted an in vivo experiment with mice. Mice that were infected with IAV were treated intranasally with PBS or BTB‐1 at 3 mpk (mg per kg). BTB‐1 treatment significantly reduced IAV titre in the lung as compared to untreated control (Figure 7A). When bodyweight was monitored, significant weight difference between PBS‐ and BTB‐1‐treated groups was not observed at 4 and 9 dpi. However, bodyweight of PBS‐treated group (CTR) was significantly lower than BTB‐1‐treated at 12 and 14 dpi (Figure 7B), indicating that BTB‐1 treatment alleviated IAV‐associated morbidity. Furthermore, BTB‐1 substantially enhanced the viability of BTB‐1‐treated mice as compared to the CTR group (Figure 7C). Thus, these results strongly suggest that inhibition of KIF18A protects mice from the morbidity and mortality induced by IAV infection.

## 4 DISCUSSION

In this study, we investigated the role of KIF18A in IAV replication. KIF18A expression increased following infection with IAV, which led us to investigate its effect on IAV replication. Our results indicated that the inhibition of KIF18A successfully suppressed IAV replication without significant cytotoxic effects. We also found that the inhibition of KIF18A impaired the nuclear export of vRNP complexes, which is a critical step in viral assembly. Therefore, targeting KIF18A has the potential to be an effective therapeutic strategy for treating IAV infection.

Viruses vigorously crosstalk with intracellular transporting systems throughout their replication process.^19^, ^21^, ^22^, ^23^, ^24^ Therefore, the inhibition of motor proteins such as KIF18A may suppress multiple steps in the viral replication process. In our results, although treatment with BTB‐1 at 1 hpi produced the strongest suppression response, treatment at 6 or 12 hpi was still able to prevent the expression of viral proteins (Figure 6A). This indicates that KIF18A plays an important role in viral replication at several different stages of the IAV life cycle. Interestingly, we observed that the time‐point and MOI for highest KIF18A expression, viral RNA expression, IAV‐induced cytopathic effect and viral RNP export were different from cell line to cell line. This is probably because each cell line has specific characteristics including cell growth rate and susceptibility rate to the IAV infection.

There are several possible mechanisms involved in the prevention of IAV replication via the inhibition of KIF18A. First, because KIF18A is a member of the motor protein kinesin family, the transport of viral RNAs and proteins may be impeded when KIF18A activity is suppressed. This could be a possible explanation for the lower expression levels of viral RNAs and proteins observed in our results (Figure 2). Second, the inhibition of KIF18A might hamper the activation of signalling pathways that are critical for IAV replication. In previous several studies, KIF18A was known to be associated with signalling pathways including Akt and metastasis‐related proteins (MMP‐7 and MMP‐9) in hepatoma cells and involved in Akt signalling pathways during human breast carcinogenesis.^35^, ^48^ In this study, we found that the inhibition of KIF18A disrupted the infection‐induced activation of signalling pathways, including AKT, p38 and SAPK that are necessary for viral replication (Figure 6). Third, kinesin family members such as KIF18A may play a role in the export of vRNP complexes, a crucial step in IAV assembly. This idea is supported by our results that inhibition of KIF18A blocked the activation of AKT, p38, SAPK and RanBP3 (Figure 6B and C), which is critical for the nuclear export of vRNP complexes.

KIF18A is also known to be critical for controlling the spindle length and aligning mitotic chromosomes at the spindle equator during cell division.^32^ Numerous studies have reported that KIF18A serves as a central regulator in cell transformation and carcinogenesis.^33^, ^34^, ^35^ For example, KIF18A is involved in several forms of cancer, including colorectal, breast and hepatocellular cancer.^33^, ^35^, ^49^ Thus, KIF18A might be a potential therapeutic target for the treatment of cancer. In this study, we provide strong evidence that KIF18A is a necessary cellular factor for IAV propagation. Although further investigations are required, KIF18A might be an advantageous factor for other viruses as well. Therefore, a single medication targeting KIF18A could have multiple applications in both cancer and infectious diseases, such as influenza.

In conclusion, the results of this study indicate that KIF18A is an important host cell factor required for successful IAV propagation. Although further investigation for the efficacy of the treatment with KIF18A inhibitor at later times post‐infection in vivo is required, inhibition of KIF18A would be an efficient therapeutic strategy for the treatment of IAV infection.

## CONFLICTS OF INTEREST

The authors declare no conflicts of interests.

## AUTHOR CONTRIBUTIONS

YC and YS designed the study and wrote the manuscript. YC, KK and JK performed experiments and analysed data. SL and Y.S supervised the study, and all authors critically revised the manuscript and approved the final version of the manuscript.

## Supporting information

Fig S1

## Data Availability

All data supporting the findings of this study are available from the corresponding author on request.
