# Supplementary figures and images for: Selective and ATP‐competitive kinesin KIF18A inhibitor suppresses the replication of influenza A virus
Source: J Cell Mol Med. 2020 Apr 6;24(10):5463–75. doi: 10.1111/jcmm.15200 (PMC7214149; doi:10.1111/jcmm.15200)

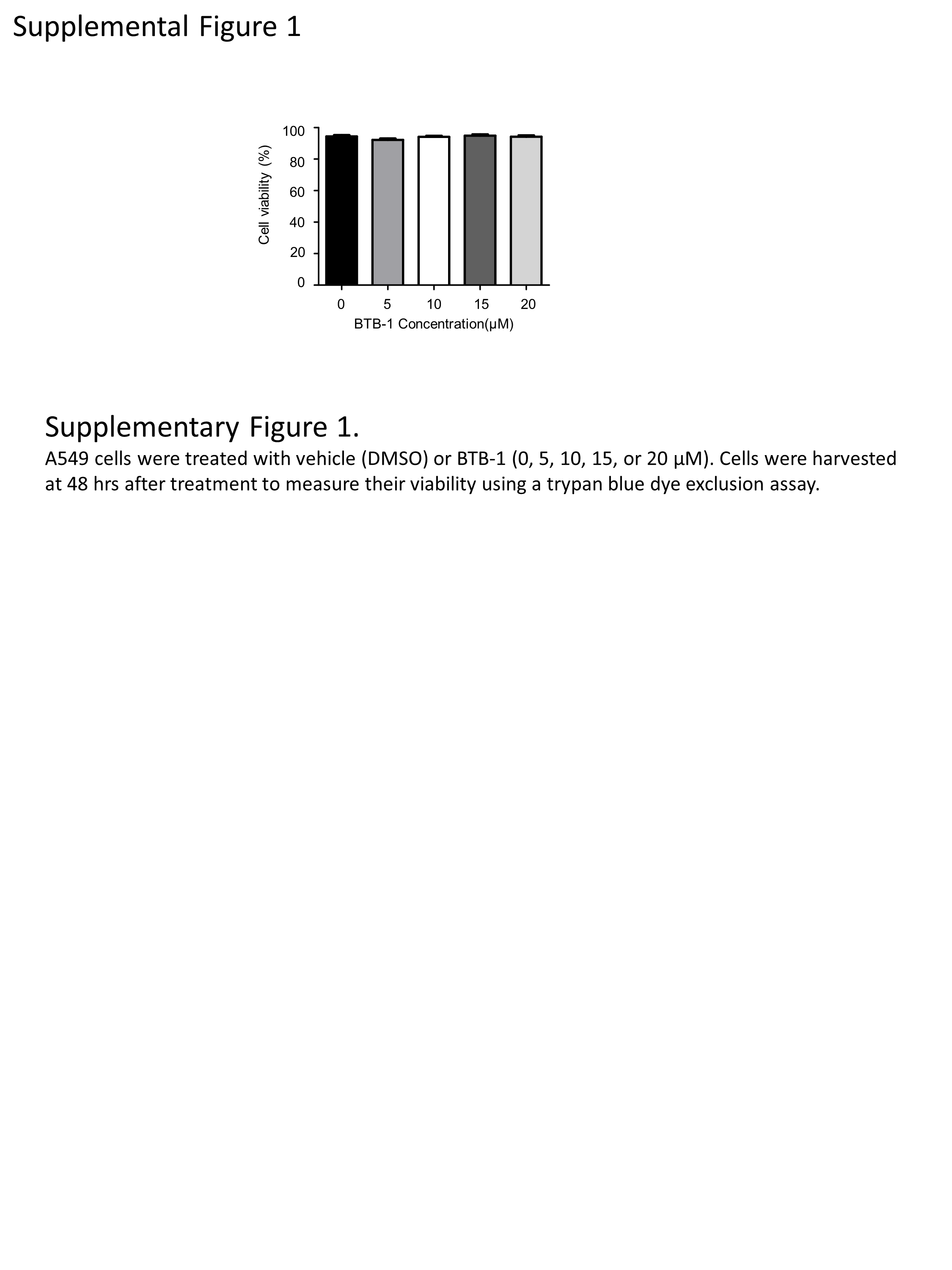

Supplement: Supplementary file 1 — Fig S1 [file JCMM-24-5463-s001.TIF]
